# Interrogating the Impact of Intestinal Parasite-Microbiome on Pathogenesis of COVID-19 in Sub-Saharan Africa

**DOI:** 10.3389/fmicb.2021.614522

**Published:** 2021-04-16

**Authors:** Dawit Wolday, Geremew Tasew, Wondwossen Amogne, Britta Urban, Henk DFH Schallig, Vanessa Harris, Tobias F. Rinke de Wit

**Affiliations:** ^1^Department of Medicine, Mekelle University College of Health Sciences, Mekelle, Ethiopia; ^2^Bacterial, Parasitic and Zoonotic Diseases Directorate, Ethiopian Public Health Institute, Addis Ababa, Ethiopia; ^3^School of Medicine, Addis Ababa University, Addis Ababa, Ethiopia; ^4^Department of Clinical Medicine, Liverpool School of Tropical Medicine, Liverpool, United Kingdom; ^5^Amsterdam University Medical Centers, Academic Medical Center, University of Amsterdam, Amsterdam, Netherlands; ^6^Department of Global Health, Amsterdam Institute of Global Health and Development, University of Amsterdam, Amsterdam, Netherlands; ^7^Global Health, Joep Lange Institute, Amsterdam, Netherlands

**Keywords:** COVID-19, microbiome, parasites, helminths, protozoa, pathogenesis, hyperinflammation, SARS-CoV-2

## Introduction

Intestinal parasitic infections affect more than 2 billion people throughout the world with disproportionately high prevalence rates in Low- and Middle-Income Countries (LMICs) (Herricks et al., 2017). Multicellular and highly complex parasites such as *Ascaris*, hook worm, *Trichuris, Enterobius* and *Schistosoma*, as well as unicellular organisms including *Entamoeba, Giardia, Toxoplasma, Cyclospora*, and *Cryptosporidium* are among major pathogens that contribute to the global intestinal parasitic disease burden.

Parasites can cause persistent infection due to their ability to resist immune-mediated expulsion by modulating the host's immune response (McSorley and Maizels, 2012; Wammes et al., 2014; Chabé et al., 2017; Burrows et al., 2019; Ryan et al., 2020). There is a complex interaction between parasites and human microbiota which can triangulate with host's immune homeostasis and host responses to bystander antigens, vaccines or other unrelated diseases, both infectious and non-communicable diseases (McSorley and Maizels, 2012; Wammes et al., 2014). Recently, the world has grappled with an unprecedented pandemic due to severe acute respiratory syndrome coronavirus 2 (SARS-CoV-2) infection that causes coronavirus disease 2019 (COVID-19) (WHO, 2020). The pathogenesis of severe disease in COVID-19 has been linked to the phenomenon of immune hyperactivation (Sinha et al., 2020; Tay et al., 2020). Here, we propose that the interplay between intestinal parasites and microbiome may have a potential direct or indirect effects on the pathogenesis of SARS-CoV-2 infection, in particular in the context of LMICs.

## Parasites Modulate Systemic Immune Responses

Though enteric parasitic infections can result in severe symptomatic disease, the majority inhabit the gut of healthy individuals and do not cause obvious signs of inflammation. Parasites have coevolved with human hosts over thousands of years resulting in persistent modulation of the immune system, through highly complex and diverse mechanisms (McSorley and Maizels, 2012; Wammes et al., 2014; Chabé et al., 2017; Burrows et al., 2019; Ryan et al., 2020).

Parasites or their products [known as Excretory Secretory (ES)] are recognized via microorganism-associated molecular patterns (MAMPs), or pathogen-associated molecular patterns (PAMPs) (McSorley and Maizels, 2012). The host's cell pattern-recognition receptors, such as toll-like receptors (TLRs), expressed in intestinal lymphoid cells are able to recognize the various molecular patterns. Overall, the gut immune response in chronic parasitic infection is largely T helper (TH) 2 in nature, characterized by activation of the innate immune system including dendritic cells (DCs), alternatively activated macrophages (AAMs), regulatory T-cells (Tregs), regulatory B cells (Bregs), eosinophils, basophils and mast cells. Cytokines produced by these cells including interleukin (IL)-4, IL-5, IL-9, IL-10, IL-13, IL-21, IL-25, IL-33, and transforming growth factor (TGF)-β will have downstream effects on the CD4+ and CD8+ T-cells of the adaptive immune system (McSorley and Maizels, 2012; Wammes et al., 2014; Chabé et al., 2017; Burrows et al., 2019; Ryan et al., 2020).

Parasite-induced immune responses are a “double-edged sword” as these may potentially be detrimental or beneficial to the host. T-cell hyporesponsiveness induced by helminths may result in increased susceptibility to infections and reduced responses to vaccines (Borkow et al., 2000; McSorley and Maizels, 2012; Wammes et al., 2014). Indeed, deworming in such conditions has been associated with improved immune responses and/or clinical outcomes (Elias et al., 2001; Wolday et al., 2002; Wammes et al., 2014). In addition, intensity of infection is one of the main factor associated with clinical presentation. In general, high intensity of infection is associated with pathology and severe form of clinical presentation. Severe disease can lead to malnutrition, malabsorption syndrome, micronutrient deficiency, anemia, stunted growth, organ damage, secondary bacterial sepsis and death (Jourdan et al., 2018). Another important factor associated with pathology is the stage of parasite life cycle. For example, hypersensitivity reaction (Loeffler syndrome) is commonly associated with the larval stages of *A. lumbricoides* and hook worms. Likewise, intestinal mucosal damage/colitis is linked to the trophozoite stage of *E. histolytica*. On the contrary, chronic persistent low intensity infection with parasites, in particular helminths, can lead to downregulation of inflammation and reduced disease severity, and deworming can aggravate the risk of inflammation associated with allergy and autoimmunity (Wammes et al., 2014). Overall the studies indicate that parasites have the capacity to suppress pro-inflammatory responses and augment regulatory pathways, and in turn may result in suboptimal immunity to infections and lower prevalence of chronic autoimmune diseases in LMICs compared with High-Income Countries (HICs) (McSorley and Maizels, 2012; Wammes et al., 2014; Chabé et al., 2017).

## Parasites Modulate Immune Responses Through Changes of Gut Microbiome Composition

In addition to the direct modulation of the host's immune response, parasites can also indirectly manipulate the immune system through changes in the microbiome. The outstanding advances made possible through the Human Microbiome Project has transformed our perception of the role of microbiome in health and disease (Turnbaugh et al., 2007). The human body harbors trillions of microbiome, which has recently been shown to play critical role in health and disease (Nicholson et al., 2012; Kåhrström et al., 2016). The composition of the microbiome varies in different individuals. Nonetheless, the majority of the species fall into the phyla of *Firmicutes, Bacteroidetes* or *Actinobacteria*. In general, a more diverse or complex microbiome at a species level is associated with healthy states, that is essential for immune homeostasis (Ivanov and Littman, 2011; Nicholson et al., 2012; Kåhrström et al., 2016; Belkaid and Harrison, 2017). On the other hand, perturbations of the gut microbiome, also known as dysbiosis, has been linked with obesity, diabetes, cardiovascular diseases, neurological disorders, cancer, inflammatory bowel diseases and autoimmunity (Kåhrström et al., 2016; Kim et al., 2017). Less diverse gut microbiome community is seen in these diseases associated with immune dysregulation, including increases in traditionally pathogenic bacteria (e.g., *Enterobacteriaceae*) and decreases in commensals (e.g., *Lactobacillus, Lachnospiraceae*, and *Ruminococcaceae*) (Nicholson et al., 2012; Kåhrström et al., 2016). Furthermore, immune dysregulation driven by dysbiosis in chronic inflammatory diseases is mainly characterized by dominant TH1 responses (Kim et al., 2017).

Studies that investigated the impact of helminths on microbiome are mainly from those conducted on experimental animal models (Reviewed in Brosschot and Reynolds, 2018). However, there is paucity of data on the interaction between parasites and human gut microbiome, with only few studies conducted to date. Individuals residing in in rural settings of LMICs with little access to sanitation often exhibit high prevalence of intestinal parasites (Herricks et al., 2017). These individuals have significantly increased diversity and composition of microbiome with potentially beneficial microbiota, although some controversy exists. The first study undertaken by Cooper et al. (2013) in Ecuador compared gut microbiome of children residing in rural area. The investigators showed that no obvious differences in microbiome composition of children with *T. trichiura* compared to those who were not infected. Nonetheless, a decreased abundance of microbiome species belonging to the *Clostridia* class of *Firmicutes*, as well as a reduction in overall microbial diversity was observed among children co-infected with both *T. trichiura* and *A. lumbricoides*. To ascertain the causal relationship between helminthic infection and microbiome diversity, characterizing the microbiome population following anthelminthic treatment is required. However, in these children infected with *T. trichiura*, anthelmintic treatment did not alter fecal microbiome composition. In the second study, Lee et al. (2014) compared microbiome composition in stool samples in helminth-infected and uninfected controls in rural Malaysia. The investigators demonstrated that a significant increase in microbiome diversity amongst individuals with helminth infection. Furthermore, they noted an increased abundance of microbiome species belonging to the *Paraprevotellaceae* family only in those infected with *T. trichiura* infection. A study done by Kay et al. (2015) have shown that Zimbabwean children infected with *S. hematobium* had a significantly higher fecal abundance of the genus *Prevetella*. Another study by Ramanan et al. (2016) also demonstrated that helminth-infected individuals in rural Malaysia harbor higher diversity of microbiota (*Faecalibacterium* and *Prevotella*) compared to helminth-negative urban residents harboring an abundance of single *Bacteroides spp*. Likewise, a study by Yang et al. (2017) in Taiwan demonstrated that infection with *Enterobius vermicularis* is associated with increased diversity of the gut microbiome. In this study, *E. vermicularis* infection was associated with a lowered relative abundance of *Fusobacteria* and an enriched proportion of *Actinobacteria*, including the probiotic *Bifidobacterium*. Treatment with mebendazole correlated with a further increase in bacterial diversity. A study by Jenkins et al. (2017) also demonstrated that microbial families belonging to *Verrucomicrobiaceae* and *Enterobacteriaceae* showed a trend toward increased abundance in helminth-infected individuals, but *Leuconostocaceae* and *Bacteroidaceae* showed a relative increase in helminth-negative and treated patients, respectively. To date, the most detailed study on the interaction of helminths and microbiome is the one undertaken by Rosa et al. (2018). This study, done across very diverse geographical regions in Indonesia and Liberia, demonstrated significant differences in composition of the microbiome community between helminth infected and uninfected individuals. Furthermore, they identified microbiome-encoded biological functions only in individuals infected with helminths.

Few studies have also documented the effect of intestinal protozoa infections on microbiota diversity in humans. Iebba et al. (2016) demonstrated that infection with *E. histolytica* and *B. hominis* were linked to eubiotic condition. Likewise, *Entamoeba* spp. (other than the pathogenic *E. histolytica*) has been found to be strongly associated with increased diversity and composition of the gut microbiota (Audebert et al., 2016). Higher diversity has also been found in individuals harboring *Blastocystis* spp. (Morton et al., 2015). These results indicate similarities between helminths and protozoa in their impact on the gut microbiome, and raise the possibility of a potentially beneficial effect of protozoa on human health. On the contrary, recent studies from endemic areas have shown that infection with the pathogenic protozoan *E. histolytica* and *G. intestinalis* alter microbiota composition to a dysbiotic state (Verma et al., 2012; Morton et al., 2015; Audebert et al., 2016; Gilchrist et al., 2016; Beatty et al., 2017). Dysbiosis in these individuals harboring *E. histolytica* were characterized by a decrease in *Bacteroides, Clostridium coccoides, C. leptum, Lactobacillu*s, *Campylobacter, Eubacterium* and *Fusobacteria*, but increase in *Bifidobacterium* spp., *Clostridiales, Ruminococcaceae*, and *Prevotella copri*.

Overall, the gut microbiome composition in individuals residing in HICs is usually less diverse than of those in LMICs, and this has been ascribed to multitude of factors, such as differences in genetic makeup, diversity of diet, rural vs. urban residence, over-usage of antibiotics and improved sanitation (Wammes et al., 2014; Blekhman et al., 2015; Martinez et al., 2015; Chabé et al., 2017). Nonetheless, in the majority of the studies noted in the above demonstrate that both helminth- or protozoa-modified microbiota diversity and shift in their composition in LMIC settings may lead to a beneficial effect on health. Whereas, disappearance of the parasites in HICs has led to increase in the prevalence of chronic inflammatory conditions.

Several reports have also demonstrated that intestinal parasites modulate the immune response through both quantitative and qualitative changes of the intestinal microbiome. Parasites can influence directly the microbiome through their antigens (or antigen products) or by interfering the homeostasis within the shared gut milieu. These studies on parasite-driven microbiota population changes and the resulting impact on immune modulation are derived mostly from experimental animal models. For example, infection of mice with the nematode *Heligmosomoides polygyrus*, altered gut microbiota led to systematic increase in pro-inflammatory cytokine type I interferon (IFN) that protected the mice against respiratory viral infection (McFarlane et al., 2017). Alternatively, parasites can alter microbiome diversity and composition via systemic immune modulation. This notion was supported by the observation that infection of mice with helminths promoted colonization of beneficial microbiota via TH2 immunity (Ramanan et al., 2016). Furthermore, a study using a primate model of idiopathic chronic diarrhea (ICD) has demonstrated that experimental administration of the helminth *T. trichiura* improved clinical symptoms of inflammation that was associated with significant changes in the composition and relative abundance of different gut bacterial species, downregulation of TH1 and the induction of TH2 immune response (Broadhurst et al., 2012). Others have also demonstrated that helminth-modified microbiota diversity in down-regulating inflammation through production of short-chain fatty acids (SCFAs) with anti-inflammatory effects (Zaiss et al., 2015; Chudnovskiy et al., 2016). SCFA production by helminth-modified microbiota likely contributes to the down-regulation of inflammation through the induction of Tregs response (Wilson et al., 2005; Arpaia et al., 2013). Importantly, infection of mice with the nematode *Nippostrongylus brasiliensis* is accompanied by significant changes of the gut microbiota composition, namely *Clostridiaceae spp*. and results in the induction of TH2 immune responses and down-regulation of TH17 immune response (Fricke et al., 2015). Indeed, the basis for the notion of helminthic therapy (Wammes et al., 2014; Ryan et al., 2020) emanated from the observed clinical improvements in subjects with chronic inflammatory conditions who received experimental infections (Giacomin et al., 2015, 2016).

Similar to the observed helminth-driven microbiota changes, parasite-modified microbiota effects on diversity and composition as well as on immune response have been documented. For example, infection of wild type mice with *Toxoplasma gondii* results in the predominance of *Enterobacteriaceae* spp., but reduction or elimination of *Bacteroidetes* and *Firmicutes* (Raetz et al., 2013). These changes in microbial composition triggers an intense TH1 immune response accompanied by high levels of IFN-⋊ production. This in turn results in severe tissue damage, including of Paneth cells and anti-microbial peptides, a pattern of immunopathology also seen in patients with inflammatory bowel disease (Frank et al., 2007). Likewise, infection with pathogenic protozoa *G. intestinalis* that alters microbiota composition to a dysbiotic state, leads to the activation of TLR signaling pathways and over-production of pro-inflammatory cytokines, including IL-1α and IL-1β (Beatty et al., 2017). On the other hand, colonization of the intestine by *Entamoeba* spp. (other than the pathogenic *E. histolytica*) and *Blastocystis* spp. has been found to be strongly associated with increased diversity and composition of a beneficial gut microbiota (Morton et al., 2015; Audebert et al., 2016; Iebba et al., 2016). These parasite-driven microbiome changes are associated with anti-inflammatory responses (Nourrisson et al., 2014).

Taken together, TH1/TH2 balances and Treg immune responses are modulated by parasite-associated modifications in the composition of the so called beneficial intestinal microbiota. However, dysbiosis of the microbial population (detrimental) has been associated with chronic inflammation. Hence, specific beneficial or detrimental outcomes following exposure to unrelated pathogen is related to specific helminth- or parasite-driven microbiome changes and the resulting down-stream immune responses.

## Immunopathogenesis of COVID-19

In December 2019, a cluster of patients with pneumonia of unknown etiology was linked to a seafood wholesale market in Wuhan, Hubei Province, China [Wuhan Municipal Health Commission (WMHC), 2019]. Subsequently, a novel coronavirus (2019-nCoV) was identified in the patients who developed severe acute respiratory infection (Zhu et al., 2020). Unprecedented rapid spread throughout the world occurred and on 30 January 2020, the World Health Organization (WHO) declared that COVID-19 is a “public-health emergency of international concern” (WHO, 2020). As of January 20, 2020, more than 96 million cases have been identified with 2 million related deaths reported (WHO Coronavirus Disease, 2020).

While most people with COVID-19 develop asymptomatic, or mild uncomplicated illness, ~14% of infected cases develop severe disease that requires hospitalization and oxygen support, and 5% require admission to an intensive care unit (Zhu et al., 2020). In severe cases, COVID-19 can be complicated by the acute respiratory distress syndrome (ARDS), sepsis and septic shock, multi-organ failure, including acute kidney injury and cardiac injury. Older age and co-morbid diseases are significant risk factors for severe disease and death.

Following entry to the respiratory tract, SARS-CoV-2 targets airway epithelial cells, alveolar epithelial cells, vascular endothelial cells and macrophages within the lung. All these cells express a receptor known as angiotensin-converting enzyme 2 (ACE2) receptor, the target receptor for receptor-binding domain (RBD) of SARS-CoV-2 (Hoffmann et al., 2020). Cell surface–associated transmembrane protease serine protease (TMPRSS2) regulate the binding of RBD to ACE2 receptor that triggers endocytosis of the SARS-CoV-2, and release of the virus into the host cells cytoplasm. Once the virus enters the cytoplasm, it will eventually hijack the host cell's machinery, initiate replication and release of new virus particles. The release of damage-associated molecular patterns along with PAMPs and MAMPs, and subsequent recognition by the PRRs of the neighboring airway cells and macrophages triggers the production of pro-inflammatory cytokines and chemokines, including IL-6, IFN-γ inducible protein (IP) 10, monocyte chemoattractant protein (MCP) 1, macrophage inhibitory protein (MIP)1-α and MIP1-β. For the majority of patients, the initial immune response is characterized by activation of innate cells and virus-specific T-cells and B-cells at the site(s) of infection and subsequent clearance of virus-infected cells and recovery. In contrast, for those who develop severe disease, the cytokines and chemokines continue to attract monocytes, macrophages, neutrophils and T cells to the site of the infection, promoting further inflammation and uncontrolled production of pro-inflammatory cytokines (Sinha et al., 2020; Tay et al., 2020). Viral pathogenesis of severe disease includes marked vascular endothelitis, thrombosis, and angiogenesis (Ackermann et al., 2020; Varga et al., 2020). Compared to mild cases, patients with severe COVID-19 have markedly increased levels of pro-inflammatory cytokines, including IL-1, IL-2, IL-6, IL-8, IL-10, IL-12, IL-17, tumor necrosis factor (TNF)-α and IFN-γ (Sinha et al., 2020; Tay et al., 2020). However, compared to non-COVID-19 ARDS, and patients with bacterial sepsis, cytokine levels are low (Sinha et al., 2020). Overall, the cytokine dysregulation associated with a COVID-19 cytokine storm is typical of a TH1 immune response. Since there are inverse regulation mechanisms between TH1 and TH2, parasite-driven microbiome changes with TH2 skewed and Treg immune responses may mute the inflammation associated with late-stage COVID-19. On the contrary, parasite-induced dysbiosis followed by TH1 responses might aggravate COVID-19 severity.

## Microbiome Composition and Diversity in COVID-19

Given the recent advent of the SARS-CoV-2 infection, the relationship between COVID-19 severity and microbiome diversity and composition is emerging. Recent reports have demonstrated that gut microbiota composition and diversity is significantly altered in patients with COVID-19 who harbored significantly dysbiotic microbiota composition when compared to healthy individuals (Reviewed in Ferreira et al., 2021). The microbiome in these patients is predominantly composed of harmful organisms, such as *Streptococcus, Rothia, Veilonella*, and *Actinomyces*, and decreased levels of beneficial symbionts, including *Agathobacter, Fusicatenibacter, Roseburia*, and *Ruminococcaceae*. In addition, a significant correlation was observed between microbial composition and COVID-19 severity; whereas microbiota that positively correlated with disease severity belonged to harmful organisms, such as *Firmicutis, Coprobacillus*, and *Clostridium* spp., beneficial microbiota, including *Alistipes ondedonkii* and *Faecalibacterium prausnitzii* negatively correlated with COVID-19 severity (Ferreira et al., 2021).

Intestinal epithelium cells, like lung epithelium cells, expresses the ACE2 receptor (Lamers et al., 2020). Intestinal epithelium cells that are in direct contact with microbiota can thus be infected with SARS-CoV-2. In addition, reports have demonstrated that gastrointestinal symptoms such as diarrhea appear to be frequent in a significant proportion of COVID-19 patients (≈20%) with a prolonged shedding of viral genome in the feces, in particular in pediatric age group (Xiao et al., 2020; Xu et al., 2020). The immune responses elicited in the intestinal epithelium are similar as in the lung (Stanifer et al., 2020). Thus, it is plausible that the intestinal immune response to SARS-CoV-2 gut may modulate microbiome composition in the same intestinal niche. This notion is supported by findings that showed that gut microbiota of patients with active SARS-CoV-2 infection of the gastrointestinal tract was characterized by enrichment of opportunistic pathogens, such as *Collinsella* spp., *Streptoccoccus*, and *Morganella* (Zuo et al., 2020a,b).

Alternatively, crosstalk between gut microbiome and the lung, also known as the “gut-lung” axis, may modulated immune homeostasis and disease development in either compartment (Enaud et al., 2020). Thus, alteration of the microbiome in the gut may influence remotely both microbial composition and immune response generated in the lung to SARS-CoV-2 and impact on COVID-19 severity.

## Impact of Parasite-Driven Microbiome Change on the Pathogenesis of COVID-19

Over many years our group has undertaken extensive investigations on the immunological profile of “apparently” healthy individuals in Ethiopia – a LIC in Africa. The immune profile of healthy Ethiopians shows evidence of chronic immune activation with significant low naïve cells but high activated memory cells, of both CD4+ and CD8+ T-cell subpopulations (Borkow et al., 2000; Hazenberg et al., 2000; Kassu et al., 2003; Tsegaye et al., 2003). Such immune characteristics in Ethiopians as compared to Europeans led us to the assumption that these could contribute to the phenomenon of milder COVID-19 symptomatology, as commonly found in SSA and elsewhere in the world (Chatterjee et al., 2020; Ssebambulidde et al., 2020). The postulation is that persistent immune activation due to continuous infections with parasites skews the immune system of populations in LMICs toward TH2 type and Treg immune responses that counteract the symptomatology associated with TH1 hyperinflammation.

Interestingly, gut microbiome disturbance has been shown to reduce host antiviral immune response, thereby aggravating lung injury caused by influenza (Ichinohe et al., 2011). In addition, it has been suggested in several reports that co-infection with helminths might increase the severity of COVID-19 in helminth-endemic areas (Bradbury et al., 2020; Gutman et al., 2020). On one hand, down regulation of the host's immune response through parasite-induced microbiome changes may result in suboptimal antiviral immunity, resulting in increased SARS-CoV-2 replication. Along these lines, it was suggested that the “potential negative effects may influence recommendations on deworming” (Hays et al., 2020). On the other hand, intestinal parasites may down-regulate the host immune and chronic inflammatory conditions, such as autoimmunity (McSorley and Maizels, 2012; Wammes et al., 2014; Chabé et al., 2017; Burrows et al., 2019; Ryan et al., 2020), potentially protecting from severe COVID-19. A recent study demonstrated that individuals with diabetes and helminth coinfection from India exhibited reduced levels of TH1, TH17 and proinflammatory cytokines, but increased TH2 and Treg immune responses (Rajamanickam et al., 2020). This effect was reversed partially following a 6-month anthelminthic treatment. In addition, several clinical trials have been carried out or are underway assessing the utility of using helminths as therapeutic agents in patients with chronic autoimmune conditions (Ryan et al., 2020).

The pathogenesis of severe COVID-19 leading to hyperinflammation resembles that of chronic inflammatory condition, such as hypertension, obesity, diabetes and inflammatory bowel diseases (Shaw et al., 2016; Fang et al., 2020). Since parasite-induced alterations of the gut microbiome has remote immunomodulatory effect on the lung, the resulting down-regulatory effects might mute the potential deleterious effects of a hyperactive immune response linked to severe COVID-19. This notion is supported by the fact that enteric helminth protect against pulmonary virus infection through interaction with microbiota (McFarlane et al., 2017). Though Bradbury et al. (2020) suggest that helminth co-infection might hasten COVID-19 severity, an alternative hypotheses suggested that helminths might indeed reduce the severity of SARS-CoV-2 infection (Fonte et al., 2020; Hays et al., 2020; Mbow et al., 2020). The later hypotheses are corroborated by the observed low fatality rate of COVID-19 in SSA setting and elsewhere in LMICs with potential high parasite loads (Fonte et al., 2020; Mbow et al., 2020). Furthermore, the proponents of this hypotheses suggest to explore the effects of experimental helminth infection (EHI) on COVID-19 severity (Hays et al., 2020). Given the fact that parasite-driven immunological responses and their reversal by deworming takes several months to exert significant immunological changes (Colombo and Grencis, 2020), we do not expect, however, a significant therapeutic impact on an acute infection with SARS-CoV-2.

**Figure 1:**
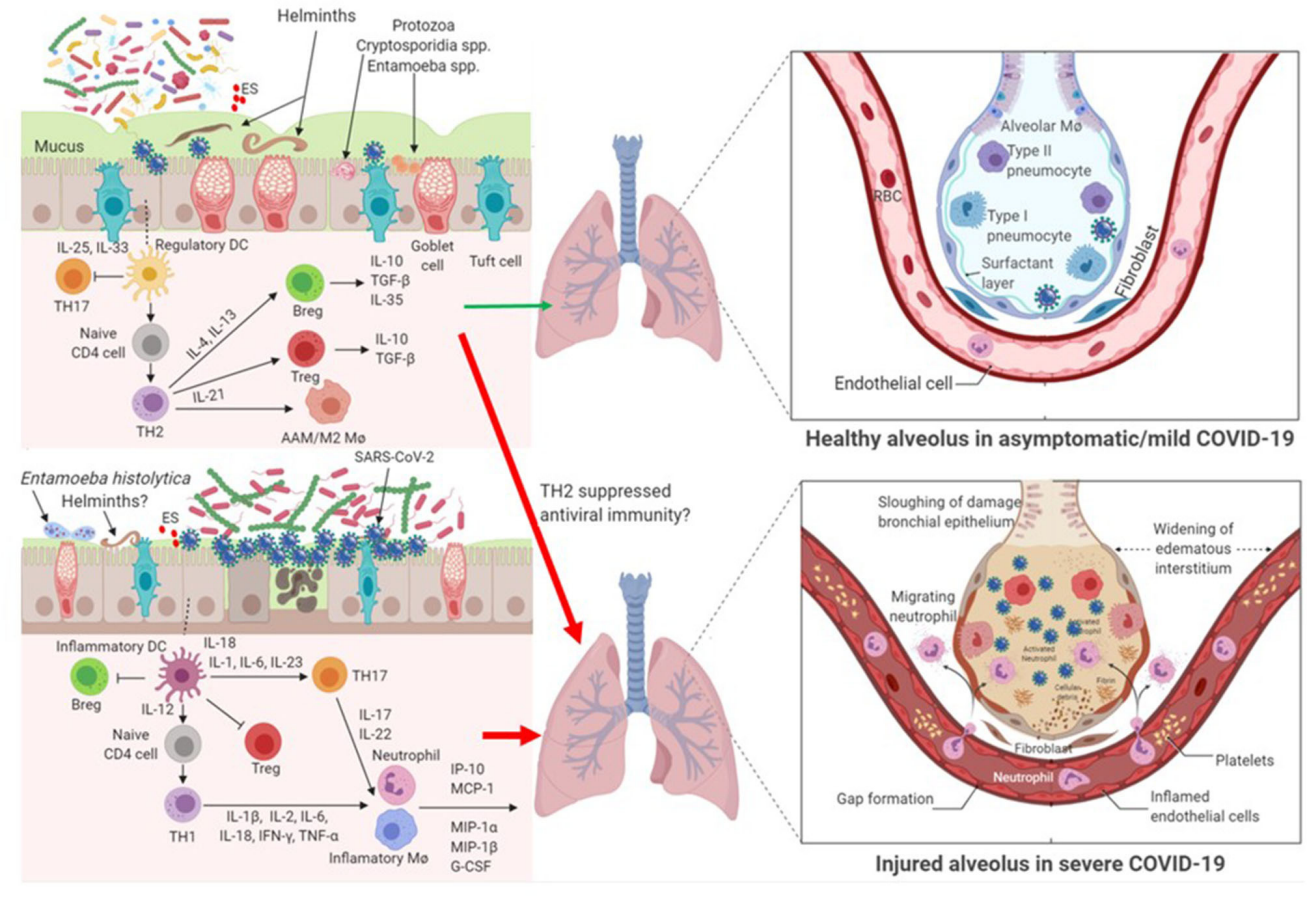
Impact of parasite-driven microbiome diversity and composition on COVID-19 severity. Certain protozoa, such as commensal Entamoeba spp. and cryptosporidium spp. and helminths that enrich the gut with the so called beneficial microbiome profile and with dominant TH2 and Treg pre-existing condition, might mute hyperactive immune response upon infection with SARS-CoV-2. In this milieu, SARS-CoV-2 replication is controlled. Another possibility is that TH2 and Treg responses might suppress anti-SARS-CoV-2 immunity and thereby hasten COVID-19 severity. On the contrary, gut dysbiosis as a result of some pathogenic protozoa, such as *Entamoeba histolytica* or helminths, or patients without parasite coinfection with underlying preexisting THl immune response might facilitate cytokine storm and aggravate COVID-19 severity. In patients with gut dysbiosis, SARS-CoV-2 replication is significantly increased. AAM/M2 Mø, alternatively-activated macrophages; Breg, regulatory B cell; ES, Excretory/Secretory parasite product; IFN, interferon; IL, interleukin; IP, interferon-γ-inducible protein; MCP, monocyte chemoattractant protein; MIP, macrophage inhibitory protein; Mø, macrophage; RBC, red blood cells; TH, T helper lymphocytes; TGF, transformation growth factor; TNF, tumor necrosis factor; Treg, regulatory T cell.

Parasites have complex interactions with the host, with different species and even different stages of parasite life cycle exerting differential immune responses in the host. Here we argue that parasitic coinfections could be either beneficial or detrimental to COVID-19 severity (Figure 1). Certain parasites, such as commensal *Entamoeba* spp. and helminths that are correlated with high bacterial diversity and dominant TH2 and Treg pre-existing profile, might mute hyperactive immune response upon infection with SARS-CoV-2. On the contrary, parasites that correlate with dysbiotic microbiota, such as those observed in pathogenic *E. histolytica* infection, reminiscent of the gut microbiome profile of individuals with chronic inflammatory conditions, might facilitate hyperinflammation and aggravate COVID-19 severity.

## Conclusion

Parasite-driven gut microbiome perturbation may alter immune response to SARS-CoV-2 infection, in particular in settings where parasitic infections are highly prevalent, such as SSA. The effect of parasite coinfection on the pathogenesis of COVID-19 remains to be elucidated. Chronic parasitic infection is common in LMICs. Such chronic infections can also indirectly manipulate the immune system through changes in the microbiota. Thus, pre-existing parasite infections may modify the host's immune response to SARS-CoV-2 with potential beneficial or detrimental effects – a “double-edged” sword. Future studies in LMIC settings should explore the effects of coinfection with parasites on the clinical course of COVID-19 outcomes. Understanding of the interplay between parasites and the microbiome and its role in the pathogenesis of COVID-19 will be important, also in light of future application of vaccine programs as well as therapeutic strategies (Margolin et al., 2020).

## Author Contributions

DW and TR conceived the idea and drafted the review. All authors contributed intellectual insights and approved it for publication.

## Conflict of Interest

The authors declare that the research was conducted in the absence of any commercial or financial relationships that could be construed as a potential conflict of interest.

The reviewer SB declared a past co-authorship with the author DW to the handling editor.
